# Altered Gut Microbial Load and Immune Activation in a *Drosophila* Model of Human Tauopathy

**DOI:** 10.3389/fnins.2021.731602

**Published:** 2021-11-02

**Authors:** Jerrik Rydbom, Halea Kohl, Vanesa R. Hyde, Kelly M. Lohr

**Affiliations:** Department of Biology, Washington and Jefferson College, Washington, PA, United States

**Keywords:** tau, *Drosophila*, gut microbiome, motility, antimicrobial peptide (AMPs), innate immune activation, tauopathies

## Abstract

Tau is a microtubule-associated protein that stabilizes the neuronal cytoskeleton. In the family of neurodegenerative diseases known as tauopathies, including Alzheimer’s disease (AD), frontotemporal dementia (FTD), and chronic traumatic encephalopathy (CTE), abnormal tau aggregation destabilizes microtubule structure, contributing to a cascade of cellular processes leading to neuronal cell death. The gut microbiome has increasingly become a target of neurodegenerative disease research since gut microbiome imbalances have been linked to protein aggregation and inflammation through a bidirectional axis linking the gut and brain. Accordingly, the present study examined tau-mediated changes to gut microbiome composition and immune activation in a *Drosophila melanogaster* model of human mutant tauopathy. Fecal deposit quantification and gastric emptying time courses suggested an abnormal food distribution and reduced gut motility in tau transgenic flies compared to controls. Tau transgenic flies also showed an increase in gut bacteria colony forming units (CFUs) from diluted fly homogenate, indicating an increased bacterial load. Finally, we showed that tau transgenic flies have a trend towards elevated systemic levels of antimicrobial peptides targeting gram-negative bacteria using qPCR, suggesting an enhanced innate immune response to bacterial insult. These data demonstrate qualifiable and quantifiable gut microbial and innate immune responses to tauopathy. Furthermore, these results provide a framework for future studies targeting the gut microbiome as a modifier of neurodegenerative disease.

## Introduction

Accumulation of the microtubule-associated protein tau is the hallmark pathology of the family of neurodegenerative diseases known as tauopathies, which includes Alzheimer’s disease (AD), frontotemporal dementia (FTD), and chronic traumatic encephalopathy (CTE). Upon hyperphosphorylation of tau, neurons undergo multiple changes leading to cell death, including alterations to cytoskeletal structure and mitochondrial function (Dias-Santagata et al., 2007; Fulga et al., 2007; Steinhilb et al., 2007; Spillantini and Goedert, 2013; Arendt et al., 2016). Due to the incidence of tauopathies in the population, a greater understanding of the effects of neurotoxic tau protein could highlight critical therapeutic pathways. Recently, the gut microbiome has become of interest due to the connectedness of the brain and gut facilitated by the gut-brain axis (D’Argenio and Sarnataro, 2019).

The human gut microbiome, colonized at birth, encompasses the collective genome of approximately one-hundred trillion microorganisms residing in the gastrointestinal (GI) tract (Ghaisas et al., 2016). These gut microbes contribute to the preservation of human health through various mechanisms including the extraction and absorption of nutrients from food, protection against pathogen overgrowth, biosynthesis of vitamins, amino acids, and peptides, interactions with the intestinal epithelium, and modulation of the immune system (Hooper et al., 2012; Zhao et al., 2015). Additionally, the microbiome is susceptible to alteration due to factors ranging from antibiotic or probiotic exposure, diet changes, environmental factors, trauma, or disease (Hill et al., 2014; Pistollato et al., 2016). Consequently, the imbalance, or disruption, of the gut microbiome has been associated with numerous pathologies (Ghaisas et al., 2016; Ma et al., 2019). Of particular interest is the implication of microbiome dysbiosis on neurological disorders and neurodegenerative diseases, as mediated by the gut-brain axis.

Using the transgenic expression of mutant human FTDP-17–associated tau, tau^R406W^, in *Drosophila*, we showed a reduced gut motility and subsequently increased gut bacterial load in aged tau transgenic flies compared to controls. We also showed an enhanced *Drosophila* innate immune response in tau transgenic flies using qPCR targeting specific antimicrobial peptide transcripts. Together, these data take advantage of the utility of transgenic *Drosophila* to show the widespread, systemic effects of tauopathy in an *in vivo* system. Furthermore, this work suggests that manipulation of the gut microbiome has potential to influence tau-mediated neurodegeneration, an important stepping stone to therapeutic approaches.

## Methods

### Drosophila Crosses

*Drosophila* stocks were obtained from Bloomington Stock Center (pan-neuronal *elav-GAL4*) and Dr. Mel Feany at Harvard Medical School (*UAS-Tau^R406W^*), respectively. Tau^R406W^ flies are referred to as “tau transgenic flies” for simplicity. All control (genotype: *elav-GAL4*/+) and tau transgenic flies (*elav-GAL4/*+*;UAS-Tau^R406W^*/+) were the progeny of controlled genetic crosses using the GAL4/UAS bipartite expression system. Prior to testing, progeny were aged for 10 days to allow for the development of neurodegeneration. All flies were crossed and aged at 25°C in an incubator programmed on a 12 h light/dark schedule in cotton-plugged vials containing commercially available *Drosophila* food (Lab Express Fly Food M). Equal numbers of male and female flies were used for each experiment unless otherwise noted in the methods.

### Fecal Deposits and Gastric Emptying Time Course

To examine gastrointestinal motility, fecal deposit counts were conducted. Vials were prepared by drawing a 2 cm × 2 cm box with a permanent marker on the outside of fly vials. The bottom of the box was drawn at the food line to reduce potentially confounding locomotor issues in tau transgenic flies. Each vial contained 7 mL of heated commercial fly food (Lab Express) mixed with 20 drops of blue food dye (Great Value brand). At 5 days post-eclosion, control and tau transgenic flies were placed into these dyed food vials and returned to the incubator for five more days of aging. At 10 days post-eclosion, flies were removed from the vials and fecal deposits within the marked boxes were quantified under a dissecting microscope. Counts were normalized to the number of flies within each vial.

To examine a time course of gastric emptying, 10-day-old control and tau transgenic flies were placed into separate blue food vials prepared as described above. Following a 24-h incubation period, flies were removed from the blue food and placed into individually labeled vials containing standard non-dyed *Drosophila* food. Flies were anesthetized at 4°C for 5 min prior to imaging. Based on previous work on anesthetization approaches, it is unlikely that the cold anesthesia approach would have preferential effects on the GI motility of control vs. tau transgenic flies (Badre et al., 2005; MacAlpine et al., 2011; Colinet and Renault, 2012; Bartholomew et al., 2015; MacMillan et al., 2016). Fly abdomens were imaged using a Leica Microsystems dissecting microscope with accompanying software and observed for the presence or absence of blue food in the abdomen at baseline and 2, 3, 4, 5, 6, and 7 h after the switch to standard non-dyed food. Flies were returned to the 25°C incubator between imaging sessions, and this process was repeated until the blue food was expelled from the abdomens of all control and tau transgenic flies. For this specific experiment, only female flies were used due to increased visibility of the abdomen.

### Agar Plate Preparation

*Acetobacter* and *Lactobacillus* are the most frequently associated genera of the *Drosophila* microbiome (Broderick and Lemaitre, 2012). Thus, two mediums were used to assess bacterial growth: MRS agar, a medium selective for Lactobacilli, and nutrient agar, a general purpose medium that cultivates a wide-range of microbes, including *Acetobacter.* MRS agar plates and nutrient agar plates were prepared using sterile techniques and poured into separate 100 mm × 15 mm culture dishes.

### 16S rDNA Sequencing

Ten-day-old control and tau transgenic flies were placed into a –20°C freezer for rapid euthanasia for 10 min. Single flies were dipped in 70% ethanol three times to reduce contamination from cuticle and allowed to dry. Flies were then homogenized individually in a sterile microcentrifuge tube containing 100 μL of autoclaved water. The resulting homogenate solution was pipetted onto two separate mediums, MRS and nutrient agar plates, and incubated at 37°C for 48 h. All plating occurred using sterile tools and techniques.

Following incubation, unique cultured bacterial colonies on each plate were identified on the basis of colony morphology and color. Representative colonies were then inoculated into liquid broth and incubated at 37°C for 24–36 h until sufficient turbidity occurred. Microbial DNA was then isolated using the Qiagen DNeasy Microbial DNA Extraction Kit according to the manufacturer’s instructions. Microbial 16S rDNA was then amplified through PCR reaction as described by the manufacturer (OneTaq, New England Biolabs) using the following cycling parameters: 5 min at 98°C, 32 1-min cycles at 94°C, 2 min at 55°C, 3 min at 72°C, 10 min at 72°C. Primer sequences were as follows: (Forward) 5′-GAGTTTGATYMTGGCTC-3′; (Reverse) 5′- GYTACCTTGTTACGACTT-3′.

PCR products were purified using the Qiagen PCR Purification Kit according to the manufacturer’s instructions; purified products were run on a 1% agarose gel and visualized with ethidium bromide to confirm successful amplification prior to sequencing. Purified 16S rDNA samples were then sent to West Virginia University’s Genomic Sequencing Core for sequencing, and the resulting sequences were identified using NCBI BLAST and Ribosome Database Project RDP^1^ analysis.

### Colony Forming Units Counts

Ten-day-old control and tau transgenic flies were euthanized by rapid cold exposure. Flies were then dipped three times in 70% ethanol solution and air dried. Individual flies were placed into sterile microcentrifuge tubes containing 200 μL sterile water and homogenized with disposable plastic pestles. Fly homogenates were briefly centrifuged to pellet the fly cuticle, which was discarded. Serial dilutions were then performed with the supernatant fly homogenate and sterilized broth, yielding 1:10, 1:100, and 1:1,000 diluted homogenates. Diluted (100 μL) and undiluted (50 μL) homogenates were spread onto MRS and nutrient agar plates. All plates were then incubated at 37°C for 48 h.

Following incubation, plates containing between 30 and 200 distinct bacterial colonies were used in the calculation of colony forming units (CFUs) per mL of plated homogenate solution, normalized to the dilution factor of the selected plate.

### qPCR

qPCR was performed as previously described (Lohr et al., 2020). Briefly, pooled RNA samples were isolated using four 10-day-old flies (two per sex) homogenized in 500 μL Trizol/Qiazol (Qiagen Cat. 73906), followed by chloroform/isopropanol extraction and centrifugation. Pelleted RNA was then washed with 70% ethanol, resuspended in DEPC water, and quantified using a NanoDrop. DNase treatment was performed using DNase I (Ambion, Life Technologies Cat. 18068-015) according to the manufacturer’s instructions. Reverse transcription was then conducted using an Applied Biosystems High Capacity cDNA RT kit (Fisher Cat. 4368814) as described by the manufacturer. The reverse transcription protocol was completed in a thermocycler for 10 min at 25°C, 2 h at 37°C, 5 min at 85°C, and held at 4°C. In a 96-well qPCR plate, 2 μL of diluted cDNA (1:2 in sterile water) was added to 14 μL of mastermix containing primer sets and 2X SYBR green mix (Fisher Cat. 4309155). The qPCR primer sets used in this study targeted the transcripts of the antimicrobial peptides attacin-A (Forward 5′-CACAACTGGCGGAACTTTGG-3′; Reverse 5′-AAACATCCTTCACTCCGGGC-3′), diptericin (Forward 5′-TACCCACTCAATCTTCAGGGAG-3′; Reverse 5′-TGGTCCACACCTTCTGGTGA-3′), and defensin (Forward 5′-AGTTCTTCGTTCTCGTGGCTA-3′; Reverse 5′-CCACATC GGAAACTGGCTGA-3′), the antifungal peptide drosomycin as a negative control (Forward 5′-CTGGGACAACGAGACCTGTC-3′; Reverse 5′-ATCCTTCGCACCAGCACTTC-3′), and the ribosomal housekeeping gene RpL32 (Forward 5′-GA CCATCCGCCCAGCATAC-3′; Reverse 5′-CGGCGA CGCACTCTGTT-3′). The plate was then run on an Applied Biosystems 7500DX qPCR machine and relative quantification (RQ) values were calculated for each transcript.

### Statistics

With the exception of the gastric emptying survival curve, all statistical analyses were performed using two-tailed *t*-tests in GraphPad Prism 5.0 software, and reported as average ± SEM. The gastric emptying curve data were analyzed for statistical significance using the Log-rank (Mantel-cox) test in GraphPad Prism.

## Results

**FIGURE 1:**
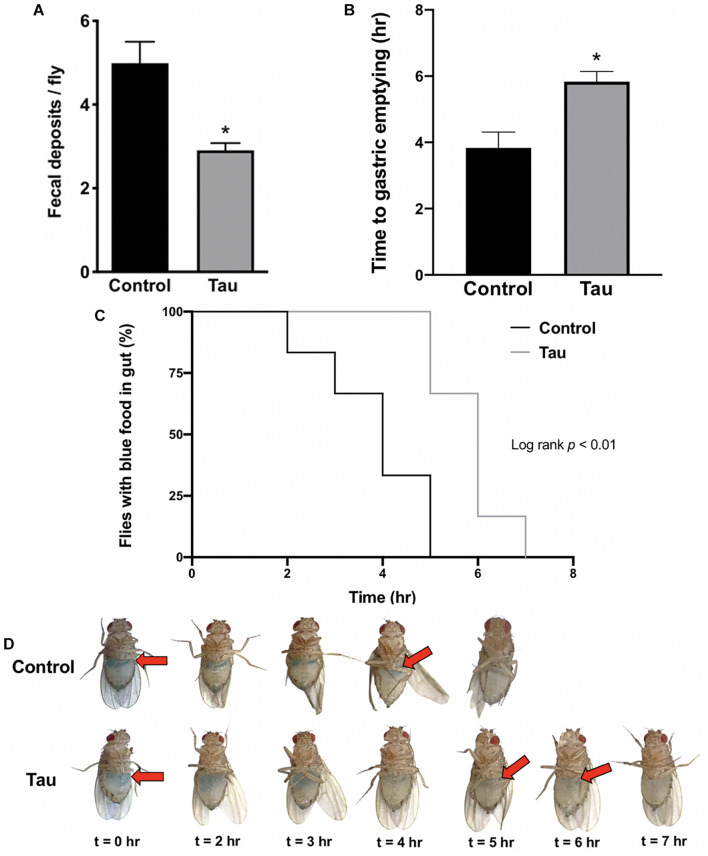
Neuronal tau expression reduces *Drosophila* gut motility. **(A)** Tau transgenic flies show reduced fecal deposits per fly (*n* = 5–7, *t*-test *p < 0.05). **(B–D)** Tau transgenic flies demonstrate an increased time to gastric emptying **(B)** (*n* = 6, *t*-test *p < 0.05), supported by the prolonged presence of blue food in the abdomen in a gastric emptying time course **(C,D)**. Data are shown as average ± SEM.

Following preliminary observations of distended abdomens and abnormal food distribution in tau transgenic flies compared to controls, a fecal deposit count was conducted to examine gastrointestinal function and motility. Tau transgenic flies showed significantly fewer fecal deposits per fly compared to controls (Figure 1A). To determine whether this reduction was due to decreased food intake or reduced gut motility secondary to neurodegeneration, a gastric emptying time course was conducted after feeding flies blue-dyed food. Images of fly abdomens (Figure 1D) showed a significantly increased time to gastric emptying for tau transgenic flies compared to controls (Figure 1B), as supported by the prolonged presence of blue food in the abdomens of tau transgenic flies (Figure 1C). These data suggest that tau transgenic flies show a reduced gut motility compared to controls. The reduced gut motility shown here is not due to neuronal protein overexpression alone as shown by a gastric emptying time course using elav-GAL4-driven eGFP transgenic flies (Supplementary Figures 1A,B). We showed no difference in gastric emptying between eGFP transgenic and control flies. Furthermore, delayed gastric emptying does not appear in all transgenic *Drosophila* neurodegeneration models. We showed no significant changes in motility in the SCA3 *Drosophila* model of spinocerebellar ataxia type 3 (SCA3; Machado-Joseph disease) (Warrick et al., 1998). However, significant reductions in gastric motility have been shown in alpha-synuclein transgenic models using both neuronal and glial drivers (Olsen and Feany, 2019), suggesting that these results may be applicable to additional proteinopathies beyond tau-mediated neurodegeneration.

Attempting to address the effect of this reduced gastric motility in tau transgenic flies, we first analyzed the taxonomic classifications of gut bacteria in control and tau transgenic flies using 16S rDNA sequencing of morphologically distinct bacterial colonies from plated fly homogenate. Sequencing analysis revealed no difference in gut bacteria classification between tau transgenic and control flies, as both genotypes showed similar bacteria profiles containing the bacterial species *Lactobacillus brevis, Lactobacillus plantarum*, and *Acetobacter pasteurianus* (Supplementary Table 1).

**FIGURE 2:**
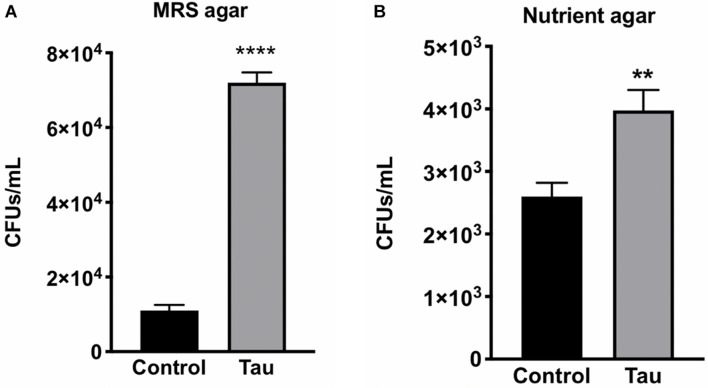
Tau transgenic flies show an increased gut bacterial load compared to controls. CFU counts of diluted tau transgenic fly homogenates were increased on both **(A)** MRS agar (*n* = 6, *t*-test ****p < 0.0001) and **(B)** nutrient agar (*n* = 5, *t*-test **p < 0.01). Data are shown as average ± SEM.

With no observable difference in the types of bacteria within gut homogenate of tau transgenic and control flies, we next examined whether there was a quantitative difference in gut bacteria between the two genotypes. To examine gut bacteria quantity, CFU counts were performed on diluted fly homogenates from control and tau transgenic flies plated on both MRS and nutrient agar. Tau transgenic flies showed significantly higher CFU counts on both MRS (Figure 2A) and nutrient agar (Figure 2B) compared to control flies.

**FIGURE 3:**
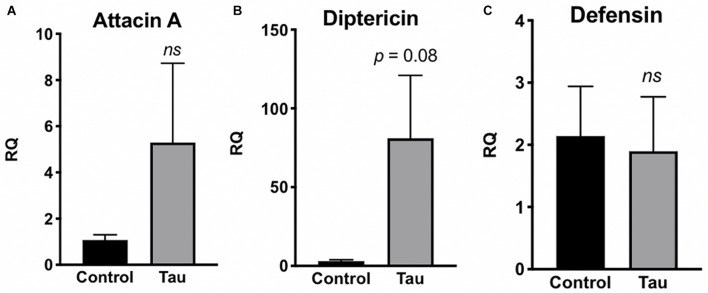
Tau transgenic flies show trends of enhanced innate immune activation to gram-negative bacteria. qPCR targeting the antimicrobial peptide transcripts attacin-A **(A)**, diptericin **(B)**, and defensin **(C)**. RQ values suggest a non-significant elevation in innate immune expression of attacin-A and diptericin, but there is no trend observed in expression levels of the antimicrobial peptide transcript defensin. (*n* = 6, *p* > 0.05). Data are shown as average ± SEM.

**FIGURE 4:**
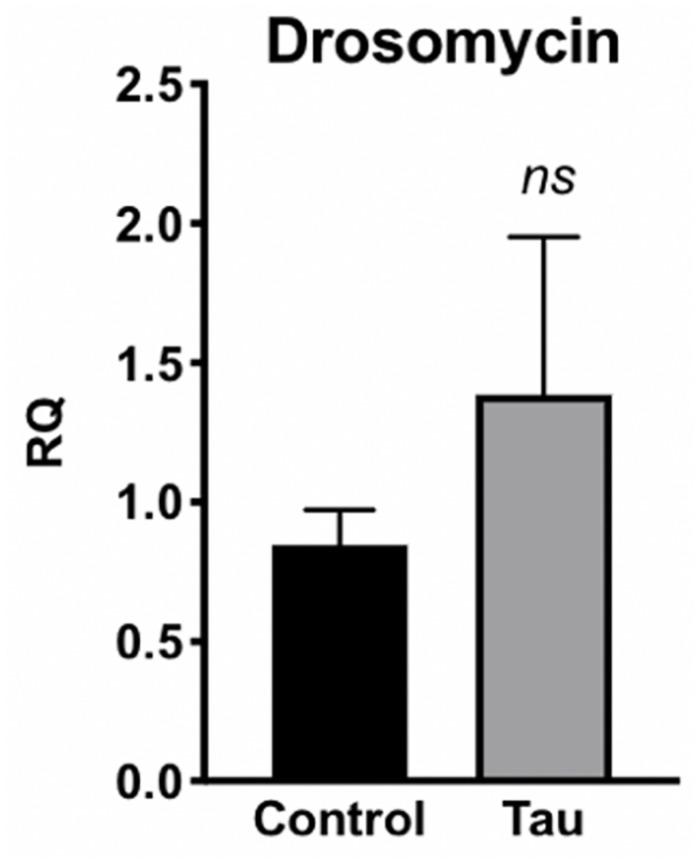
Tau transgenic flies show no significant difference in antifungal AMP transcript levels. qPCR targeting the antifungal peptide transcript drosomycin showed no significant difference in expression levels between tau transgenic and control flies (*n* = 6, *p* > 0.05). Data are shown as average ± SEM.

Due to this increased bacterial load in the guts of tau transgenic flies, we next examined whether tau transgenic flies also displayed an innate immune response. In response to bacterial insult, the Toll and Imd NF-kB signaling pathways of the *Drosophila* innate immune system are activated, regulating the production of antimicrobial peptides (AMPs) which target and degrade the cell walls of bacteria, facilitating microbial death (Hultmark, 2003; Hanson and Lemaitre, 2020). Accordingly, expression levels of AMP transcripts are often used to monitor innate immune activity. To measure innate immune activation for tau transgenic flies compared to controls, we performed qPCR on control and tau transgenic fly homogenate using specific primers for the AMP transcripts attacin-A, diptericin, and defensin, and the antifungal transcript drosomycin as a negative control. Tau transgenic flies showed a trend toward increased expression for the AMP transcripts attacin-A (Figure 3A) and diptericin (Figure 3B), both gram-negative response AMPs, compared to controls. No differences were seen in transcript levels of defensin, a gram-positive response AMP (Figure 3C) or the antifungal transcript drosomycin, as expected (Figure 4).

## Discussion

This study examined the effects of transgenic tau expression on gastrointestinal function and motility, the gut microbiome, and innate immune activation in *Drosophila melanogaster.* Tau transgenic flies showed significantly reduced gastric motility compared to controls. Widespread neurodegeneration in tau transgenic *Drosophila* is well characterized (Wittmann et al., 2001; Dias-Santagata et al., 2007; Fulga et al., 2007; Khurana et al., 2012; Lohr et al., 2020). Thus, it is not surprising that degeneration of neurons innervating the enteric nervous system of the gut would also occur in this aging fly model. This degeneration may contribute to the slowed gut motility and subsequent systemic changes shown in this study.

It has been suggested by multiple groups that *Drosophila* serve as an ideal model system for a microbial assessment due to the ease of environmental control and relative simplicity of the microbiota (Kenmoku et al., 2017; Trinder et al., 2017; Clark and Walker, 2018; Selkrig et al., 2018). While the *Drosophila* microbiota has 1–30 species dominated by *Lactobacillus* and *Acetobacter* (Blum et al., 2013; Erkosar et al., 2013; Chaston et al., 2014), it is estimated that the human GI microbiota is far more complex, with as many as 500 different bacterial species present (Quigley, 2013, 2017). Furthermore, both fly and human GI tracts also have similar structural anatomy, innervation, and function (Pitsouli et al., 2009; Apidianakis and Rahme, 2011).

Changes to gastrointestinal composition and function has become a point of interest in many types of neurodegeneration. Clinically, it is known that Parkinson’s disease patients demonstrate delayed gastric emptying and reduced gut motility (Hardoff et al., 2001), and these gastrointestinal symptoms have been supported by deposits of alpha-synuclein within the enteric nervous system (Beach et al., 2010; Gelpi et al., 2014). α-Synuclein transgenic flies also show constipation as shown by a similar assay to the one used here (Olsen and Feany, 2019). Thus, the reduced GI motility and increased bacterial load shown in the present study may apply to other types of degeneration. Although digestive disorders, including irritable bowel syndrome, have also been associated with AD and related dementias (Liao et al., 2020), tau-mediated enteric nervous system changes remain poorly understood in tauopathies (Chalazonitis and Rao, 2018; Derkinderen et al., 2021). As such, the present study provides a potential role of tau in enteric nervous system degeneration, perhaps contributing to gastrointestinal symptoms, including reduced gut motility. Finally, there are additional variables to consider when interpreting the gut motility data from the current study. It should be noted that the retention of the blue-dyed food shown in the tau transgenic fly gut could be due to a leaky gut barrier and not just slowed motility of the tract. While it is also difficult to entirely eliminate potential differences in food consumption between control and tau transgenic flies, the gastric emptying time course suggests that tau transgenic flies eat similar amounts of food compared to controls as shown by similar starting levels of gastric filling.

Although 16S rDNA sequencing of gut homogenate showed no difference in gut bacteria classification between the genotypes, CFU counts revealed a significantly increased bacterial load in tau transgenic flies. It is possible that this increased bacterial quantity is due to the slowing of gastric emptying in the tau transgenic flies. Mammalian studies have shown that alterations to gastric motility can significantly alter proportions of bacterial types, contributing to gut dysbiosis (Sun et al., 2019). While the *Drosophila* gut has multiple differences in comparison to the mammalian GI tract, its structure and function are similar (Trinder et al., 2017). Thus, slowed gastric motility may be a contributing factor to the increased bacterial load seen in the tau transgenic flies.

Despite lacking the adaptive immunity characteristic of vertebrates, *Drosophila* has proven an important model in examining the interplay between gut microbiome homeostasis and innate immunity (Ryu et al., 2010). AMP expression increases in response to systemic bacterial infection as a way to destroy pathogens (Hanson and Lemaitre, 2020). In the current study, elevated AMP levels may be a systemic response to the enhanced bacterial load in tau transgenic flies (Figure 2). Furthermore, these AMPs may also be altering the relative levels of bacteria in the fly gut. Tau transgenic flies showed a trend toward elevated attacin-A and diptericin levels, two AMPs responsive to gram-negative bacteria such as *Acetobacter* (Imler and Bulet, 2005). This increased expression of gram-negative AMP responders may be contributing to the relatively low nutrient agar CFUs compared to that of MRS agar, which grows gram-positive *Lactobacillus* bacteria (Figure 2). This suggests that the elevated gram-negative responsive AMPs may be working to reduce the gram-negative bacterial load in tau transgenic flies, whereas less pathogenic types of gram-positive bacteria may be less regulated. As expected, expression levels of the antifungal peptide transcript drosomycin were unchanged.

These results are in line with the endotoxin hypothesis of neurodegeneration where endotoxin, a lipopolysaccharide (LPS) in the outer layer of gram-negative bacteria, contributes to neuronal dysfunction, particularly during infection and inflammation (Brown, 2019). Endotoxin treatment induces microglial activation, memory dysfunction, and neuronal changes in rodents and has been shown to promote formation of several neuropathologies, including aggregation of tau, amyloid β, and alpha-synuclein (Lee et al., 2008; Gardner et al., 2016; Kim et al., 2016). Further adding to the gut-brain connection, some neurodegenerative diseases consistently present altered gut microbiomes compared to controls. For example, in Parkinson’s disease, the gut microbiome is significantly altered and has been associated with gram-negative endotoxin-producing bacteria, including *H. pylori* (Scheperjans et al., 2015; Shen et al., 2017). These data suggest that gram-negative bacteria may contribute to neuronal dysfunction in some of these disease states.

The present study is significant in that it provides evidence for tau-mediated alterations to gut motility and microbiome composition. Furthermore, this work extends the links between AMP activity, innate immune mechanisms, and tau-mediated neurodegeneration. However, the precise manner through which AMPs may contribute to neurodegeneration remains poorly understood (Hanson and Lemaitre, 2020). As such, future studies should examine neurodegeneration and microbiome composition using germ-free *Drosophila*, reintroduction of specific gut bacterial species, antimicrobial conditions, or AMP transgenic flies. Further studies may also examine the gain or loss of innate immune signaling activity through an analysis of Toll and Imd receptor expression or analyze neuronal AMP expression through reporter assays. Such routes will undoubtedly strengthen our understanding of the interplay between AMP activity, innate immune signaling, and neurodegeneration.

## Data Availability Statement

The raw data supporting the conclusions of this article will be made available by the authors, without undue reservation.

## Author Contributions

KL, JR, HK, and VH completed the experiments, analyzed the data, and edited the manuscript. JR and KL wrote the manuscript. All authors contributed to the article and approved the submitted version.

## Conflict of Interest

The authors declare that the research was conducted in the absence of any commercial or financial relationships that could be construed as a potential conflict of interest.

## Publisher’s Note

All claims expressed in this article are solely those of the authors and do not necessarily represent those of their affiliated organizations, or those of the publisher, the editors and the reviewers. Any product that may be evaluated in this article, or claim that may be made by its manufacturer, is not guaranteed or endorsed by the publisher.

## Footnotes

1
https://rdp.cme.msu.edu/classifier/classifier.jsp

## Supplementary Material

Supplementary Figure 1 Neuronal overexpression of eGFP and SCA3 shows no significant differences in gastric emptying at 10 day. eGFP and SCA3 transgenic flies show no difference in time to gastric emptying compared to controls **(A)** (*n* = 6, ANOVA, *p* > 0.05). These results are supported by similar retention of blue food in the abdomen in a gastric emptying time course **(B)**. Data are shown as average ± SEM.

Supplementary Table 1 Bacteria identified by 16S rDNA sequencing in fly homogenate.
