# Supplementary material for: Altered Gut Microbial Load and Immune Activation in a Drosophila Model of Human Tauopathy
Source: Front Neurosci. 2021 Nov 2;15:731602. doi: 10.3389/fnins.2021.731602 (PMC8597733; doi:10.3389/fnins.2021.731602)
Supplement: Supplementary Figure 1 — Neuronal overexpression of eGFP and SCA3 shows no significant differences in gastric emptying at 10 day. eGFP and SCA3 transgenic flies show no difference in time to gastric emptying compared to controls (A) (n = 6, ANOVA, p > 0.05). These results are supported by similar retention of blue food in the abdomen in a gastric emptying time course (B). Data are shown as average ± SEM. [file Data_Sheet_1.docx]

**SUPPLEMENTARY MATERIAL**


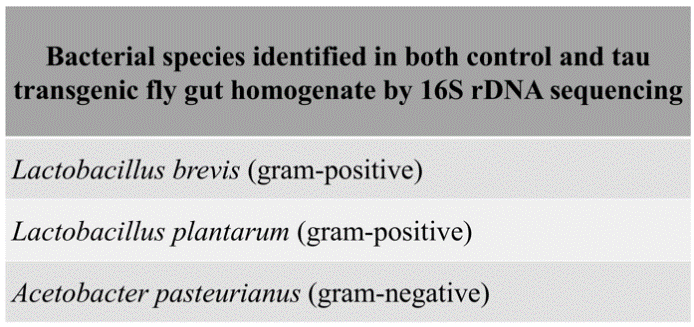


**Supplementary Table 1. Bacteria identified by 16S rDNA sequencing in fly homogenate.**


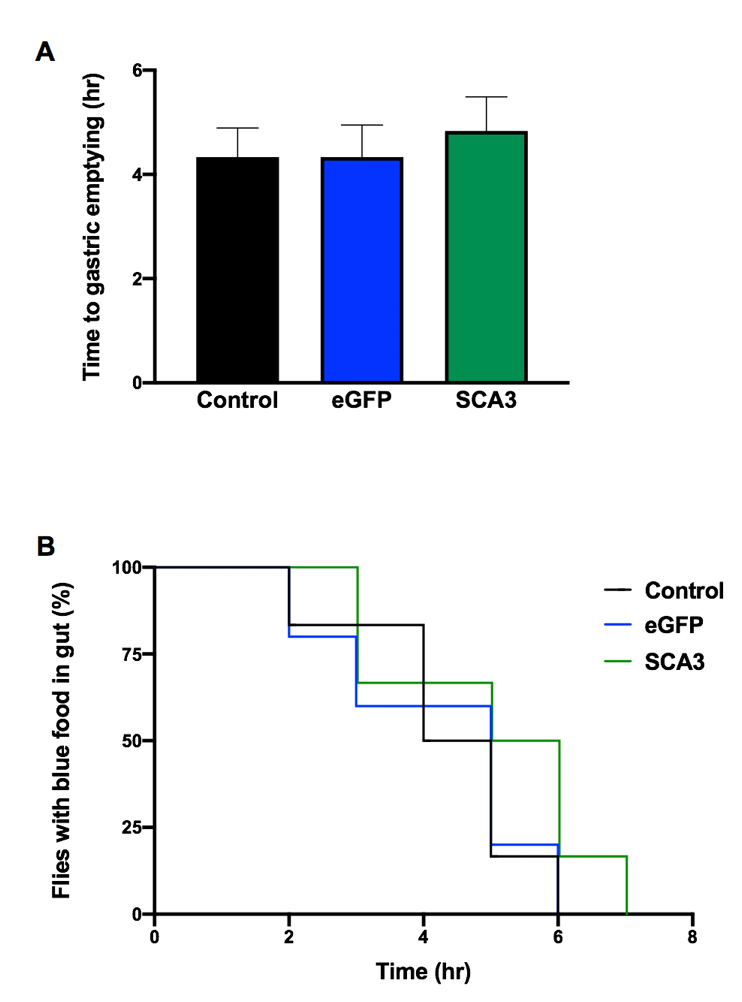


**Supplementary Figure 1. Neuronal overexpression of eGFP and SCA3 shows no significant differences in gastric emptying at 10 d.** eGFP and SCA3 transgenic flies show no difference in time to gastric emptying compared to controls (A) (n=6, ANOVA, p>0.05). These results are supported by similar retention of blue food in the abdomen in a gastric emptying time course (B). Data are shown as average ± SEM.
